# Timely HAART initiation may pave the way for a better viral control

**DOI:** 10.1186/1471-2334-11-56

**Published:** 2011-03-01

**Authors:** Paola Paci, Federico Martini, Massimo Bernaschi, Gianpiero D'Offizi, Filippo Castiglione

**Affiliations:** 1 Biomedical University Campus, via Alvaro del Portillo 21, 00128 - Rome, Italy; 2 National Institute for Infectious Diseases "Lazzaro Spallanzani", I.R.C.C.S., 00149 Rome, Italy; 3 Institute for Computing Applications "Mauro Picone", National Research Council, 00185 Rome, Italy

## Abstract

**Background:**

When to initiate antiretroviral therapy in HIV infected patients is a diffcult clinical decision. Actually, it is still a matter of discussion whether early highly active antiretroviral therapy (HAART) during primary HIV infection may influence the dynamics of the viral rebound, in case of therapy interruption, and overall the main disease course.

**Methods:**

In this article we use a computational model and clinical data to identify the role of HAART timing on the residual capability to control HIV rebound after treatment suspension. Analyses of clinical data from three groups of patients initiating HAART respectively before seroconversion (very early), during the acute phase (early) and in the chronic phase (late), evidence differences arising from the very early events of the viral infection.

**Results:**

The computational model allows a fine grain assessment of the impact of HAART timing on the disease outcome, from acute to chronic HIV-1 infection. Both patients' data and computer simulations reveal that HAART timing may indeed affect the HIV control capability after treatment discontinuation. In particular, we find a median time to viral rebound that is significantly longer in very early than in late patients.

**Conclusions:**

A timing threshold is identified, corresponding to approximately three weeks post-infection, after which the capability to control HIV replication is lost. Conversely, HAART initiation occurring within three weeks from the infection could allow to preserve a significant control capability. This time could be related to the global triggering of uncontrolled immune activation, affecting residual immune competence preservation and HIV reservoir establishment.

## Background

The question of when antiretroviral therapy has to be initiated remains a challenging issue. Recent studies show that the early immune response to HIV-1 infection is likely to be an important factor in determining the clinical course of disease [1]. The first weeks following HIV-1 transmission are extremely dynamic. They are associated with rapid damage to generative immune cell micro-environments and with immune responses that partially control the virus. Following HIV-1 infection, the virus first replicates locally in the mucosa and then is transported to draining lymph nodes where further amplification occurs. This initial phase of infection, until the systemic viral dissemination begins, constitutes the eclipse phase [1]. In general, there is an exponential increase in plasma viremia with a peak 21-28 days after infection. By this time, significant depletion of mucosal CD4^+^T cells has already occurred. Around the time of peak viremia, patients may become symptomatic and reservoirs of latent virus are established [1,2].

The "window of opportunity" between the infection and peaking of viremia, prior to massive CD4^+ ^T cell destruction and the establishment of viral reservoirs, seems to be a narrow but crucial period in which an antiretroviral therapy can control viral replication, prevent an extensive CD4^+ ^T cell depletion from occurring and curb generalized immune activation. Thus, thwarting HIV replication by introducing HAART in the early phases of infection could have a substantial impact on the whole disease course. In particular, suggested factors that may contribute to the observed better viral control after treatment interruption in very early treated patients are [3]: *i*) early arrest of viral escape, leaving the virus vulnerable; *ii*) preservation or even enhancement of the immune response resulting from the early clearing of antigen; *iii*) prevention of the establishment of a pool of HIV-specific memory CD4 T cells thus leaving fewer target cells available for viral infection.

An ideal clinical model aimed at addressing such issue should compare a number of patients treated starting on different times: from very early to very late. Besides the ethical issues, it is rather difficult to collect enough patients to significantly represent the whole spectrum of possible HAART initiation timings. As a matter of fact, a practical clinical model would compare very early to late-treated patients. While informative on the overall role of HAART timing on disease course, this approach would not allow to verify if there are events in the early infection influenced by the starting time of HAART that affect directly and decisively the course of the disease.

We have already shown in [4-6] that an agent-based model of HIV-1 infection could be a valuable tool for the study of the AIDS disease progression and treatment. The computerized simulation allows us to track the effect of HAART timing on the progression of the disease.

The aim of the present work is to verify the effect of HAART timing on subsequent events. Indeed, both a clinical model and a computational simulation show that a late initiation of treatment affects HIV-1 replication control. Interestingly, the *in silico *model identifies a significant three-week time threshold as the "ultimate" time point beyond which the decisive HIV-induced damages already occurred, affecting the whole disease course.

In a previous work [4], we analyzed clinical data of patients initiating HAART within six months from infection (i.e., we called that *early *phase) and performed computer simulations to predict the differences in viral rebound at therapy interruption between those patients and subjects initiating therapy during the chronic phase (i.e., we called that *late *phase, corresponding to initiating six or more months after infection). Our conclusion was that early initiation of therapy does not prolong the disease-free period when compared to a treatment started during the late phase. However, other studies suggest that an earlier initiation is preferable [7-9]. This motivated us to better identify the meaning of early initiation. In the present article, we extend the analysis in [4] to get a more complete picture. We analyze clinical data of *very early *patients (i.e., treated before seroconvertion) against late-treated patients.

## Methods

### Clinical studies

We analyzed the results of clinical studies performed at the Clinical Department of the National Institute for Infectious Disease "L. Spallanzani" in Rome.

A first group of eleven patients (9 male and 2 female) were diagnosed HIV-1 positive between year 1998 and 2006. All patients initiated HAART within 14 days from diagnosis, during the very early phase of infection (see Table 1). The very early phase was defined as having a negative or indeterminate western blot for HIV-1 antibodies in combination with a positive test for either p24 antigen or a detectable HIV-1 RNA concentration. Those patients were treated with zidovudine/lamivudine (CBV) in combination with either the reverse transcriptase inhibitor efavirenz (EFZ) or one protease inhibitor lopinavir/ritonavir (KAL) or indinavir (IDV). Because anaemia and neutropenia were diagnosed, in two cases CBV has been substituted with lamivudine (3TC) and staduvine (D4T). All those patients underwent a therapy cycle for 2 ± 1 years and remained off HAART for about 48 weeks.

**Table 1:** Very early subjects with an immediate treatment before seroconversion.

ID	Therapy^† ^therapy	Elapsed days ^‡ ^therapy	At diagnosis*		4 weeks after**		8 weeks after**	
			CD4	vRNA	CD4	vRNA	CD4	vRNA
Pz4	CBV, IDV	14	603	16000	800	< 50	792	6000
Pz34	CBV, EFZ	18	370	280000	647	640	815	< 50
Pz36	CBV, EFZ	15	402	14000		< 50	599	< 50
Pz38	CBV, EFZ	2	543	3500000		1600	770	220
Pz41	CBV, EFZ	12	568	1300000	1549	< 50	857	< 50
Pz42	CBV, EFZ	8	234		1083	2300	657	< 50
Pz47	CBV, IDV	4	753	8802	615	220	743	< 50
Pz51	CBV, EFZ	10	530	91972	655	< 50	810	< 50
Pz67	CBV, KAL	0	797	21675	884	98	932	< 50
Pz83	CBV, KAL	18	426	> 500000	770	2349	1524	1037
Pz113	CBV, KAL	3	261	65716	422	660	460	302

Clinical information about the eleven patients selected at the Clinical Department of the National Institute for Infectious Disease "L. Spallanzani" in Rome. All subjects received HAART within six months from primary infection.

^†^CBV, Combivir (AZT Zidovudine plus 3TC Lamivudine); IDV, Indinavir; EFZ, Efavirenz; KAL, Kaletra (lopinavir/ritonavir). ^‡ ^Days elapsed from diagnosis (enrollment) to initiation of HAART. *CD4 and CD8 are per microlitre of plasma, viremia is per millilitre of plasma. ** 4 weeks after treatment interruption.

The second group is made up by twenty-two patients (21 male and 1 female) enrolled in the program between year 1998 and 2005. Patients in this group underwent HAART during the early phase of HIV-1 infection. In particular, they started HAART about 20 days after treatment diagnosis (see Table 2). Early patients were defined as having documented seronegative HIV-1 antibody test within the previous 6 months; acute symptomatic seroconversion illness; evolving HIV-specific antibody response by ELISA; positive HIV-DNA PCR in PBMC. Those patients were treated with three different drugs (in the majority of cases zidovudine (AZT) plus 3TC plus a protease inhibitor. Further details can be found in Table One of [4]. All those patients underwent a therapy cycle for 3 ± 1 years and remained o HAART for about 88 weeks.

**Table 2:** Early subjects with an immediate treatment of acute HIV-1 infection.

ID	Therapy^†^	Elapsed days ^‡^	Days on therapy	Days out therapy	At diagnosis*			At first interruption		
					CD4	CD8	vRNA	CD4	CD8	vRNA
Pt 03	D4T,3TC,IDV	14	1775	27	653	1659	75	809	459	5794
Pt 05	AZT,3TC,IDV	61	1657	27	444	633	100	867	476	< 50
Pt 29	AZT,3TC,NFV	14	1069	28	1103	1964	31	741	844	< 50
Pt 33	AZT,3TC,IDV	66	925	28	424	991	0.13	458	474	147
Pt 35	AZT,3TC,EFV	1	1041	30	522	1205	78	919	607	496
Pt 37	AZT,3TC,EFV	0	1371	790	545	1233	3.9	901	384	< 50
Pt 58	AZT,3TC,EFV	3	442	27	768	1632	50	1451	1580	< 50
Pt 06	AZT,3TC,IDV	32	1620	1908	882	4146	4.1	754	726	< 50
Pt 18	AZT,3TC,IDV	26	635	213	1319	1810	15	1718	825	< 80
Pt 24	AZT,3TC,IDV	3	1231	542	507	3162	78	513	1067	< 50
Pt 31	AZT,3TC,EFV	19	1102	1654	322	243	490	1154	1165	< 50
Pt 41	AZT,3TC,EFV	12	705	1837	568	278	130	1326	940	< 50
Pt 45	AZT,3TC,EFV	5	488	55	307	1032	130	872	506	< 50
Pt 53	AZT,3TC,Lop/rit	16	503	179	341	779	18.9	545	529	150
Pt 04	AZT,3TC,IDV	6	1561	2155	603	1288	1.6	1047	1015	88
Pt 19	AZT,3TC,IDV	13	1480	1640	1338	716	10	1113	447	< 50
Pt 28	AZT,3TC,NFV	7	325	70	281	860	190	863	485	< 80
Pt 32	AZT,3TC,EFV	15	1384	669	409	1448	4.3	960	331	< 50
Pt 72	AZT,3TC,LPV	7	318	343	827	386	19	1005	751	< 50
Pt 81	AZT,3TC,NVP	33	745	317	412	264	32.5	522	461	68
Pt 85	AZT,3TC,Lop/rit	46	717	717	326	669	50	855	1436	< 50
Pt 92	3TC,Lop/rit,TNF	0	455	331	616	3774	46.3	1027	913	< 50

Clinical information about the twenty-two patients selected at the Clinical Department of the National Institute for Infectious Disease "L. Spallanzani" in Rome. All subjects received HAART within six months from primary infection.

^†^D4T, staduvine; 3TC, lamivudine; IDV, Indinavir; AZT, Zidovudine; NFV, nelflnavir; EFV, Efavirenz; NVP, nevirapine; TNF, Tenofovir; Lop, lopinavir; Rit, Ritonavir; LPV (KAL), Kaletra (lopinavir/ritonavir). ^‡ ^Days elapsed from diagnosis (enrollment) to initiation of HAART. *CD4 and CD8 are per microlitre of plasma, viremia is per millilitre of plasma.

The third group consists of twenty-one patients (12 male, 9 female). They started HAART during the chronic phase of infection defined as suggested by the guidelines [10]. In particular, they started HAART about 3.5 years after treatment diagnosis (see Table 3). Their CD4 count at initiation was 400 ± 150 per microlitre of plasma. The range of calendar year for starting HAART among those patients was 1998 ± 3. All those patients underwent a therapy cycle for 4 ± 2 years and remained off HAART for about 41 weeks. The Ethical Committee of the "L. Spallanzani" Institute approved the study and the patients gave a written informed consent.

**Table 3:** Late subjects with deferred treatment of acute HIV-1 infection.

ID	Therapy^†^	Elapsed days ^‡^	Days on therapy	Days out therapy	At diagnosis*			At first interruption		
					CD4	CD8	vRNA	CD4	CD8	vRNA
Pt 07	AZT, 3TC	300	1303	606	463	2498	34000	614	3101	9154
Pt 12	D4T, 3TC, SQV	26	1117	89	422	754	62000	601	948	< 50
Pt 16	AZT, ddC, Rit	9	1803	66	116	1170	990000	483	1165	< 50
Pt 17	AZT, 3TC	49	1872	412	51	1155	110000	693	1359	150
Pt 18	AZT, ddl	268	1615	558	340	994	35000	989	1172	< 50
Pt 19	AZT, 3TC	393	819	206	559	1230	70319	616	825	< 50
Pt 20	AZT, 3TC	15	2503	21	235	877	230000	566	1246	< 50
Pt 22	D4T, 3TC	0	1807	64	290		140000	1334		< 50
Pt 23	AZT, 3TC	483	2109	248	409	734	47000	686	572	< 50
Pt 25	D4T,3TC, IDV	22	1661	625	383	809	37000	896	1208	< 50
Pt 26	AZT, 3TC	38	841	1150	540	1256	16000	719	640	< 50
Pt 27	AZT,3TC	10	1885	50	332		2800	636		< 50
Pt 28	AZT,3TC,EFV	0	3131	373	556		1900	752		< 50
Pt 29	AZT,3TC,Lop/rit	21	1950	69	483		150000	1233		100
Pt 31	AZT,3TC,IDV	0	1255	109	574	1625	7900	813	640	< 50
Pt 32	AZT,3TC,IDV	0	1472	160	393		1335	1235		< 50
Pt 33	D4T, 3TC	407	523	66	479	494	473	705	450	< 50
Pt 34	ddl, D4T, NVP	13	2207	54	493	1628	230000	907	943	< 50
Pt 38	AZT,3TC,LPV	0	1460	270	481	1156	20000	534	964	< 50
Pt 39	AZT,3TC,NVP	64	2734	767	539		41000	859		< 50
Pt 41	AZT,3TC,Lop/rit	62	428	89	200		135000	848		< 50

Clinical information about the twenty-one patients selected at the Clinical Department of the National Institute for Infectious Disease "L. Spallanzani" in Rome. All subjects received HAART during chronic phase of infection.

^†^AZT, Retrovir (Zidovudine); 3TC, Epivir (Lamivudine); D4T, Zerit (Staduvine); SQV, Saquinvir (Invirase); Zalcitabina (ddC, Hivid); Rit, Ritonavir (Norvir); ddl, Didanosine (Videx); IDV, Indinavir (Crix-ivan); EFV, Efavirenz; NVP, Nevirapine (Viramune); LPV (Lop), Lopinavir; ^‡ ^Days elapsed from diagnosis (enrollment) to initiation of HAART. *CD4 and CD8 are per microlitre of plasma, viremia is per millilitre of plasma.

### Plasma HIV-1 determination

Plasma HIV-1 RNA levels were determined by a second-generation assay based on nucleic acid sequence based amplification (NASBA), for samples collected until 2001 and by the branched-chain DNA assay (Versant HIV RNA test, Version 3.0, lower limit of quantification 50 copies/*ml*; Bayer Diagnostics, Milan, Italy) from 2001 until 2008.

### Computational model

The current version of the model we employ derives from an early simulator that has been quite extensively described in previous publications [11,12]. Recently it has been specialized to simulate the HIV-1 infection [6] and the effects of antiretroviral therapy [4].

Briefly, it resorts to bit strings to represent "binding sites" of cells and molecules, as for example lymphocyte receptors, MHC, antigen peptides and epitopes, immunocomplexes, etc. [13]. The model includes the major classes of cells of the lymphoid lineage (T helper lymphocytes, cytotoxic T lymphocytes, B lymphocytes and antibody-producer plasma cells) and some of the myeloid lineage (macrophages and dendritic cells). These entities are individually represented. In contrast to cells, cytokines like interleukin-2 are represented in terms of concentrations and their dynamics described by a parabolic partial differential equation plus a degradation term accounting for the finite half-life [5,14]. Modeling features of the HIV infection include HIV replication inside infected lymphocytes, T production impairment; specific response against HIV strains and HIV mutation.

The simulated life cycle of the virus is represented by the following stages: 1) the virus infects CD4^+ ^T cells, macrophages, dendritic cells; 2) reverse transcriptase copies the viral single stranded RNA genome into a double-stranded viral DNA. The viral DNA is then integrated into the host chromosomal DNA; 3) the virus remains at rest until an event activates the transcription; 4) the replicating virus buds from the cell membrane. Fully assembled virions are then able to infect other cells to restart the life cycle. HAAR effects are modeled as follows: Reverse transcriptase inhibitors block reverse transcriptase enzymatic functions and avoid completion of synthesis of the double-stranded viral DNA thus preventing HIV-1 from replicating (i.e., it prevents the virus in stage 1 from reaching stage 2); Protease inhibitors prevent viral replication by inhibiting the activity of HIV-1 protease, an enzyme used by the virus to cleave nascent proteins for final assembly of new virions (i.e., it prevents virus assembly in stage 4). Further details and parameter settings of the simulations can be found in the Additional file 1.

For what concerns the setting of the parameters related to the therapy, we performed computer simulations in which we fixed the immunological parameters at the time of therapy initiation on the basis of the average values measured in patients *in vivo*: 5.8 ± 0.2 RNA copies/*ml *(in logarithmic scale), 870 ± 50 CD4 cells/*μl *and 430 ± 50 CD8 cells/*μl*. For all simulations we applied a one-year course of HAART. Further details on the tuning of the simulation parameters can be found in the Additional file 2.

## Results

We analyze virological data from HIV patients treated during the very early, early and late phase of infection and compare them with computer simulations.

**Figure 1:**
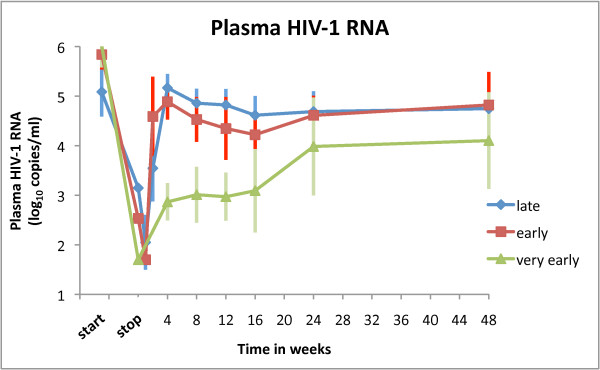
**Plasma HIV-1 RNA load**. The mean plasma HIV-1 RNA load versus time in weeks after interruption of HAART for patients classified in late (21 subjects), early (22 subjects) and very early (11 subjects) groups. Error bars show standard deviations. The features of the three clinical settings are given in section Patients and Methods.

In Figure 1 clinical data of all three analyzed groups is shown altogether. In a point-by-point comparison we find no statistical difference in viral rebound between early and late-treated patients (P ≥ 0.05, Mann-Whitney U two-tailed test) confirming the results of [4]. In addition, we observe that a difference does exist for very early initiation of therapy (P < 0.05, Mann-Whitney U two-tailed test).

In the present work we extend the simulations of [4] to include the new clinical settings corresponding to a very early initiation of therapy. In particular, the very early simulation settings correspond to a beginning of the therapy within the first week whereas the late settings correspond to initiating therapy between week five and six from infection.

**Figure 2:**
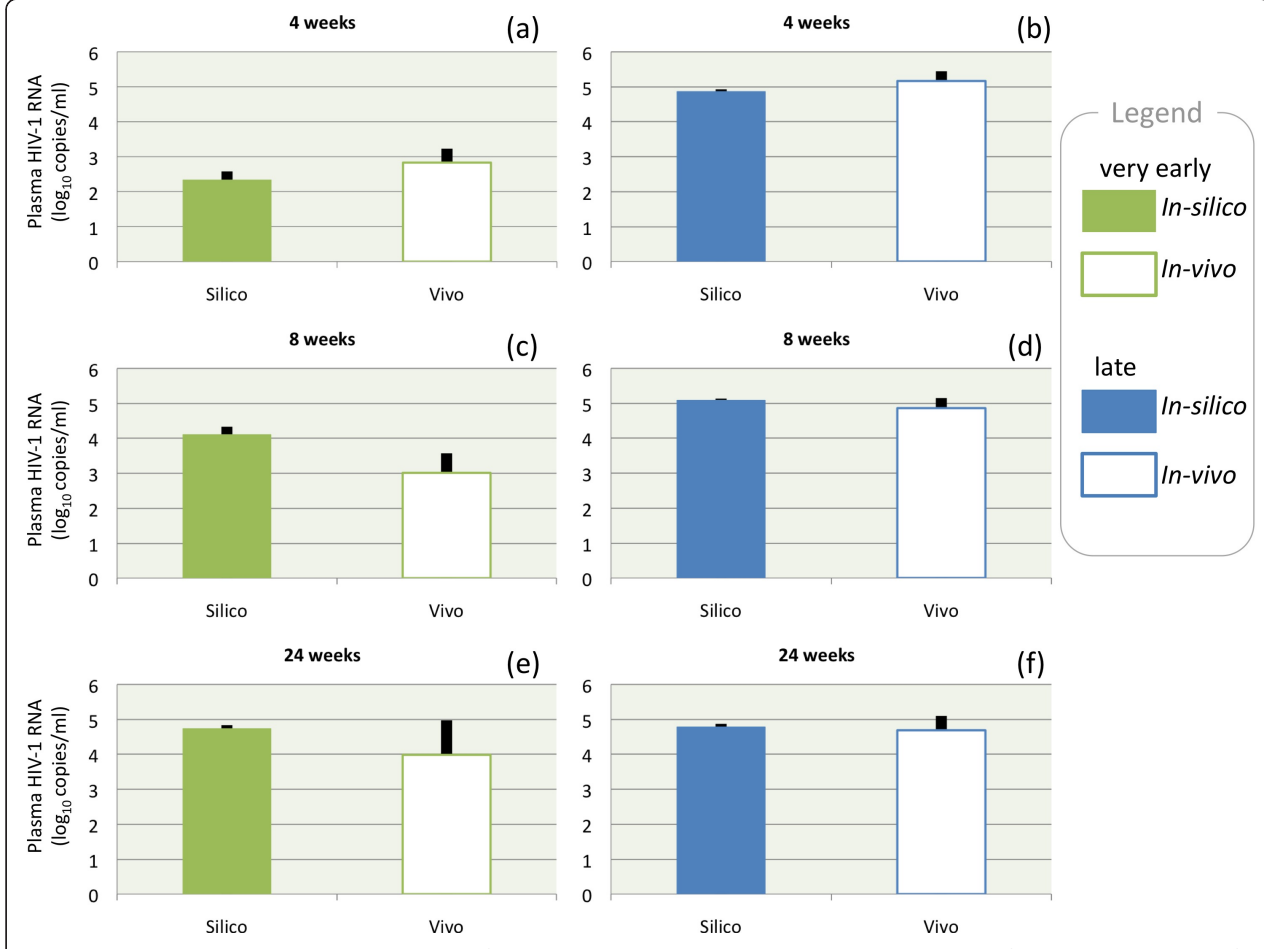
**Virological rebound**. Virological rebound after 4 weeks (top), 8 weeks (middle) and 24 weeks (bottom) from therapy interruption for two groups: very early (green boxes) that started HAART before seroconversion and late (blue boxes) that started HAART during chronic phase of primary HIV-1 infection. Filled boxes represent *in silico *data (resulting from three thousands runs) whereas empty boxes correspond to *in vivo *data. We have calculated the Mann-Whitney U test statistics for assessing whether the two independent samples (*in silico and in vivo*) come from the same distribution. In all cases we did not find a significant difference (P ≥ 0.05, Mann-Whitney U two-tailed test). Black lines indicate standard deviations. Information on parameter settings for the simulations can be found in the Additional file 1.

Figure 2 summarizes data of virological rebound (averages) after therapy interruption at different time points (4, 8 and 24 weeks) for very early and late patients for both clinical (empty boxes) and simulation data (filled boxes). Firstly, the figure shows that clinical and simulation data are in good agreement (differences are not statistically significant: P ≥ 0.05, Mann-Whitney U two-tailed test). Secondly, the difference in plasma HIV-1 RNA (copies/ml) between very early and late-treated patients decreases with increasing time from therapy interruption. Panel (a) shows a difference of about two logs with panel (b) for both clinical data and simulations. These differences vanish after 24 weeks from therapy interruption (cfr. panel (e) and (f)). The overall message is that a delay in the initiation of therapy reduces the chances of maintaining a therapy effect at discontinuation.

**Figure 3:**
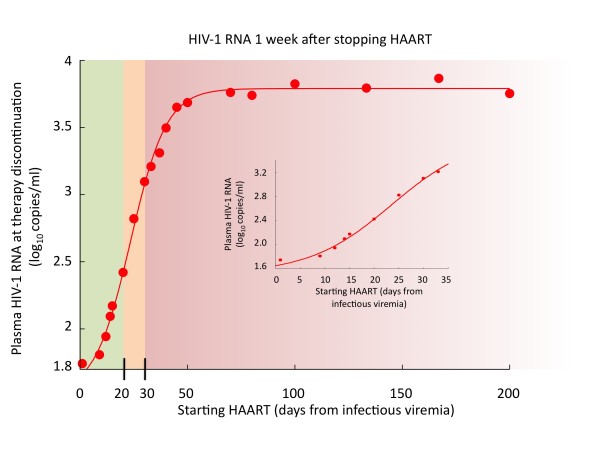
**Virological rebound after 1 weeks**. Virological rebound at 1 week after therapy interruption for different starting time points of HAART (*t_s_*). The red dots represent the results of thousands simulations and the fitting line is given by the Richards' curve in equation 1. Standard deviation is about 0.1 log_10_(*copies/ml*) for all points. Inset plot is a zoom for *t_s _*< 30 days. Parameters of the fitting curve are: *a *= 1.52, *k *= 3.79, *d *= 0.12, and $t_{s}^{*}$ = 23.50.

In order to provide a more precise estimate of the time "limit" beyond which the benefit of an early initiation of therapy vanishes, we use the simulation to investigate the influence of HAART initiation time (*t_s_*) on the viral rebound. The corresponding results are shown in Figure 3. The virological rebound at *one week *after therapy interruption as a function of *t_s _*is presented. We observe that there are two regimens, one for *t_s _*< 20 days and one for *t_s _*> 30 corresponding to what clinicians call respectively *best controllers *(with undetectable HIV RNA levels) and *rebounders *(whose HIV viremia load returns, approximately, to the pre-HAART level).

The points in Figure 3 fit pretty well a generalized logistic function (i.e., the Richards' curve, [15]) describing the growth of viremia as a function of *t_s_*,

$$V(t_{s})=a+\frac{k-a}{1+e^{-d(t_{s}-t_{s}^{*})}}$$

where the parameter *k *is the carrying capacity or the upper asymptote, *a *is the lower asymptote, *d *is the growth rate, and $t_{s}^{*}$ is the time of maximum growth. By moving the time of the measurements beyond one week after therapy interruption, the resulting data still fit the same *V *(*t_s_*) but with a greater *a*, a smaller d and a greater $t_{s}^{*}$. In particular the limit for d going to zero, of *V *(*t_s_*) is (*a *+ *k*)/2 may lead to the deceiving conclusion that there is no window of opportunity because the viral rebound is independent from *t_s_*.

**Figure 4:**
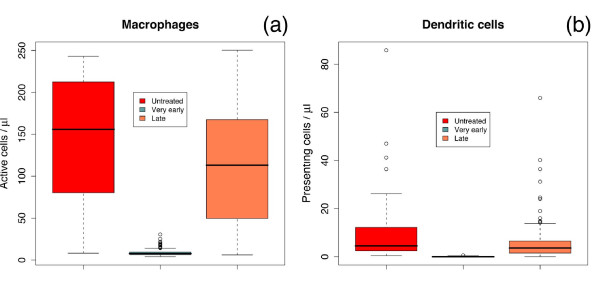
**Inflammatory response**. Cell counts of active macrophages (a) and dendritic cells presenting viral proteins on class II MHC molecules (b) show that, in the simulation, the late-treated case is comparable to the control case (untreated) whereas the very early case stands on its own. This suggests that the set up of an infiammatory state affects the viral rebound at therapy discontinuation. Counts are taken at week 8 for all groups. Therapy for very early started within the first week and for the late started at about week 6.

With respect to $t_{s}^{*}$, the value of~ 23:5 days points to the early inflammation as a critical phase of the disease. To bring into focus this facet, we compare two simulated "markers" of the inflammation state in untreated (control case), very early and lately treated simulated patients (see Figure 4). These virtual markers are given by the cell counts of active macrophages (a) and dendritic cells presenting viral proteins on class II MHC molecules (b). We observe that the late-treated case is comparable to the control case (untreated) whereas the very early stands on its own. This observation suggests that it is the activation of the immune system through the set up of an in ammatory state that has to be blamed for the increased viral rebound for *t_s _*>$t_{s}^{*}$.

Figure 4 shows with clarity that very early initiation of the treatment can down-regulate the immune activation, hence limiting viral replication and spread. Interestingly, this view is supported by the observation that HIV triggers the immune activation directly (e.g., HIV gene products can induce the activation of lymphocytes and macrophages as well as the production of pro-inflammatory cytokines and chemokines [2]) or indirectly (e.g., sustained antigen-mediated immune activation occurs in HIV-1-infected patients due also to other viruses like the cytomegalovirus or the Epstein-Barr virus [2]). In both case, the result is a high level of pro-inflammatory cytokines, such as tumor necrosis factor alpha, interleukin 6 and interleukin 1 beta, right from the early stages of HIV-1 infection [2].

## Discussion

Recent analysis (performed by Fiebig et al. [16]) of samples from individuals that have been infected by HIV-1 has revealed that patients can be categorized into six stages on the basis of a sequential gain in positive HIV-1 clinical diagnostic assays (viral RNA measured by PCR, p24 and p31 viral antigens measured by enzyme-linked immunosorbent assay (ELISA), HIV-1-specific antibody detected by ELISA and HIV-1-specific antibodies detected by western blot, [16]). Patients progress from acute to early chronic infection at the end of stage V, approximately 100 days following infection, as the plasma viral load begins to stabilize.

With respect to the study conducted by clinical data analysis and computer simulation described so far, we identify three regimens, as highlighted in Figure 3. These can be paralleled to Fiebig et al. stages [16]. In particular, we observe that patients treated with HAART in very early stages of the infection (stage I-III) are likely to better control the viremia after treatment interruption [3]. If therapy starts in the acute phase (stage V-VI) then the action of the drug foils the immune response and, as a consequence, at the end of the therapeutic period, the virus rebounds undisturbed.

**Figure 5:**
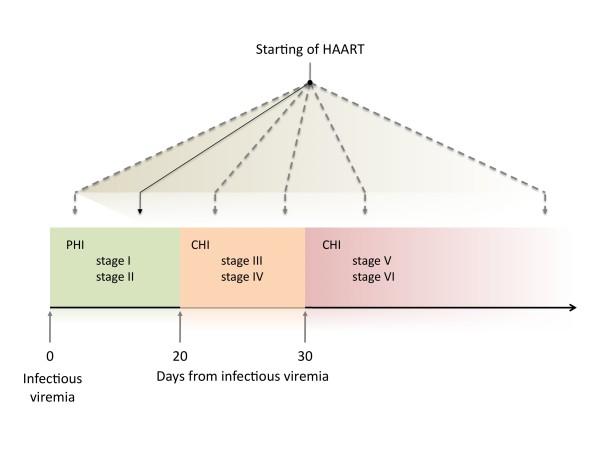
**Phase diagram**. Schematic diagram of the importance of an early initiation of HAART with respect to the progression of HIV markers according to the staging in [16].

These considerations are summarized in Figure 5 where we draw a schematic picture of the importance of an early initiation of HAART with respect to the progression of HIV markers according to Fiebig's et al. stages. In particular we identified the "window of opportunity" corresponding to stages I-III, that is, the first three weeks from primary HIV-1 infection. Patients receiving therapy in this narrow period are likely to turn out to be best controllers. Probably, the massive immune activation in the early stage of the disease favors the virus, as it finds more host target cells to exploit for replication. In point of fact, the ensuing massive depletion of CD4+ T cells in mucosal lymphoid tissues, can result in the disruption of the mucosal barrier in the gut. This barrier prevents the translocation of the intestinal flora from the gut to the systemic immune system restricting it to the lamina propia and the mesenteric lymph nodes [2]. HIV-1 infection is indeed associated with a significant increase of plasma lipopolysaccharide levels that is an indicator of microbial translocation, directly correlated with measures of immune activation.

## Conclusions

A number of studies indicate that interfering with HIV replication by starting the therapy in the early phases of the infection could have a deep impact on the whole disease course. However, HAART is costly, it is onerous for both patient and health care provider, and often brings adverse effects. Its clinical benefit must therefore be weighed against its burden.

In the present study, we resorted to a computer model to study the dynamics of the plasma viral load after prolonged treatment interruption in two groups of *in silico *patients: those who initiate HAART very early and those who start it lately. We evaluated the model comparing the results to clinical data. We found that an opportunity time-window exists for the initiation of HAART (roughly within three weeks before the establishment of viral reservoirs), in which the therapy can control viral replication, preventing generalized immune activation and extensive CD4+ T cell depletion.

## Availability and requirements

An educational version of the immune system simulator is available on our website:

- Name: C-ImmSim

- Home page: http://www.iac.rm.cnr.it/~filippo/C-ImmSim.html

- Operating system(s): Linux, Unix Mac OS X, Windows

- Programming language: C

- Licence: C-ImmSim is available under a LICENSE AGREEMENT that needs to be signed: http://www.iac.rm.cnr.it/~filippo/how-to-get-cimmsim_files/LicenseAgreement.pdf

## Competing interests

The authors declare that they have no competing interests.

## Authors' contributions

FC and PP designed and performed research; all authors wrote the paper. FM and GDO provided the clinical data. All authors read and approved the final manuscript.

## Pre-publication history

The pre-publication history for this paper can be accessed here:

http://www.biomedcentral.com/1471-2334/11/56/prepub

## Supplementary Material

Additional file 1 **Mathematical model details**. This file lists all the interactions between cells and molecules considered in the model and an accurate description of the parameter setting. Click here for file

Additional file 2 **Parameters tuning for the simulation of HIV infected virtual patients**. This file reports details of the parameter setting and tuning. Click here for file

## References

[B1] McMichael AJ Borrow P Tomara GD Goonetilleke N Haynes BF The immune response during acute HIV-1 infection: clues for vaccine development Nat Rev Immunol 2010 10 11 23 10.1038/nri2674 20010788 PMC3119211

[B2] Appay V Sauce D Immune activation and inflammation in HIV-1 infection: causes and consequences J Pathol 2008 214 231 241 10.1002/path.2276 18161758

[B3] Steingrover R Pogány K Garcia EF Jurriaans S Brinkman K HIV-1 viral rebound dynamics after a single treatment interruption depends on time of initiation of highly active antiretroviral therapy AIDS 2008 22 1583 1588 10.1097/QAD.0b013e328305bd77 18670217

[B4] Paci P Carello R Bernaschi M D'Offizi G Castiglione F Immune control of HIV-1 infection after therapy interruption: immediate versus deferred antiretroviral therapy BMC Infect Dis 2009 9 172 10.1186/1471-2334-9-172 19840392 PMC2771028

[B5] Paci P Castiglione F Bernaschi m Baldazzi V A discrete/continuous model of anti-HIV response and therapy IEEE Computer Society, Digital library Proceedins UKSIM 2008 481 486

[B6] Castiglione F Poccia F D'Offizi G Bernaschi M Mutation, Fitness, Viral Diversity, and Predictive Markers of Disease Progression in a Camputational Model of HIV Type 1 infection AIDS Res Hum Retrovirus 2004 20 12 1314 1323 10.1089/aid.2004.20.1314 15650424

[B7] Ahdien-Grant L Yamashita TE Phair JP Detels R Wolinsky SM When to Initiate Highly Active Antiretroviral Therapy: A Cohort Approach Am J Epidemiol 2003 157 738 746 10.1093/aje/kwg036 12697578

[B8] Autran B Carcelain G Li TS Blanc C Mathez D Positive Effects of Combined Antiretroviral Therapy on CD4^+ ^T Cell Homeostasis and Function in Advanced HIV Disease Science 1997 277 112 116 10.1126/science.277.5322.112 9204894

[B9] Yamashita TE Phair JP Muñoz A Margolick JB Detels R Immunologic and virologic response to highly active antiretroviral therapy in the Multicenter AIDS Cohort Study AIDS 2001 15 735 745 10.1097/00002030-200104130-00009 11371688

[B10] DHHS Guidelines for the use of Antiretroviral Agents in HIV-1 Infected Adults and Adolescent 2008 http://aidsinfo.nih.gov date last accessed

[B11] Celada F Seiden PE A computer model of cellular interaction in the immune system Immunology Today 1992 13 2 56 62 10.1016/0167-5699(92)90135-T 1575893

[B12] Bernaschi M Castiglione F Design and implementation of an immune system simulator Computers in Biology and Medicine 2001 31 5 303 331 10.1016/S0010-4825(01)00011-7 11535199

[B13] Farmer JD Packard NH Perelson AS The immune system, adaptation and machine learning Physica D 1986 22 187 204 10.1016/0167-2789(86)90240-X

[B14] Baldazzi V Paci P Bernaschi M Castiglione F Modeling lymphocytes homing and encounters in lymph nodes BMC Bioinformatics 2009 10 387 10.1186/1471-2105-10-387 19939270 PMC2790470

[B15] Richards FJ A flexible growth function for empirical use J Exp Bot 1959 10 290 300 10.1093/jxb/10.2.290

[B16] Fiebig EW Wright DJ Rawal BD Garrett PE Schumacher RT Peddada L Heldebrant C Smith R Conrad A Kleinman SH Busch MP Dynamics of HIV viremia and antibody seroconversion in plasma donors: implications for diagnosis and staging of primary HIV infection AIDS 2003 17 1871 1879 10.1097/00002030-200309050-00005 12960819

