# Supplementary material for: Timely HAART initiation may pave the way for a better viral control
Source: BMC Infect Dis. 2011 Mar 1;11:56. doi: 10.1186/1471-2334-11-56 (PMC3058048; doi:10.1186/1471-2334-11-56)
Supplement: Additional file 1 — Mathematical model details. This file lists all the interactions between cells and molecules considered in the model and an accurate description of the parameter setting. [file 1471-2334-11-56-S1.PDF]

Supplementary material S.1

Material and Methods

1 The C-ImmSim model of the immune system

The model of immune system response we employ, C-ImmSim, has been quite extensively described in previous works (see references in the article). It makes use of bit strings to represent the "binding site" of cells and molecules as for example lymphocyte receptors (T lymphocytes receptor TCR, B lymphocytes receptor BCR), Major Histocompatibility Complexes MHC, antigen peptides and epitopes, immunocomplexes, *etc.*. The affinity among the entities is a function of the Hamming distance between these bit strings, that is, the affinity increases as the complementarity fit of the bit strings. CImmSim includes the major classes of cells of the lymphoid lineage (T helper lymphocytes or TH, cytotoxic T lymphocytes or CTL, B lymphocytes and antibody-producer plasma cells, PLB) and some of the myeloid lineage (macrophages, MA, and dendritic cells, DC). All these entities interact each other following a set of "rules" that describe the different phases of the recognition and response of the immune system against a pathogen.

A single lymph node of a vertebrate animal is mapped onto a three-dimensional ellipsoid lattice. The primary lymphoid organs thymus and bone marrow are modelled apart: the thymus is implicitly represented by the positive and negative selection of immature thymocytes before they get into the lymphatic system, whereas the bone marrow generates already mature B lymphocytes. Hence, on the lattice there are only immunocompetent lymphocytes.

The virus is represented by two binary strings (each l bits long), one corresponds to the B cell epitope (*i.e.* the BCR's binding site) and the other one to the T cell epitope (*i.e.* the MHC class I and II's binding site). The code allows the specification of an arbitrary number of epitopes and peptides. Each time step of the simulation corresponds to eight hours of "real life".

The interactions among the cells determine their functional behaviour (the complete list of interactions is reported in table 1). Interactions are coded as probabilistic rules that define the transition of each entity from a state to another. Conversely to classic cellular automata models, there is no correlation among entities residing on different sites at a fixed time step and the deterministic character of automata dynamics is replaced by a stochastic behavior. However, at the end of each time step entities diffuse from site to site introducing spatial correlations. Each interaction requires cell entities to be in one specific out of a set of possible states (naïve, active, resting, duplicating, etc.). Once this condition is fulfilled, the interaction probability depends on the effective binding between receptors. In particular we define as *affinity function* a monotonic (*i.e.* exponential) function of the Hamming distance in the space of the bit-strings.

Unlike the majority of the immunological models, CImmSim has an additional level of description, representing the intracellular processes of antigen digestion and presentation. Both the *cytosolic* and *endocytic* pathways are implemented. Respectively, the endogenous antigen is fragmented and combined with MHC class I molecules for presentation on the cell surface to CTLs' receptors, whereas the exogenous antigen is cut into smaller parts (*i.e.,* peptides) which are then bound to MHC class II molecules for presentation to the THs' receptors (table 2).

While the virus and the antibodies are uniquely represented (*i.e.* they are agents like the cells), for other molecules with small molecular weight, like interleukins or chemokines, only their spatial concentration is represented. The corresponding dynamics is modeled by the following parabolic partial differential equation that describes a uniform diffusion process with the addition of a degradation term that takes into account the finite half-life of molecules:

$$\frac{\partial c}{\partial t} = D\nabla^2 c - \lambda c + s(x,t) \tag{1}$$

where $c = c(x,t)$ is the concentration of chemokines, $s(x,t)$ is the source term (*e.g.* macrophages), D is the diffusion coefficient and λ is the half-life. We assume $D = 3000 \mu m^2/min$ and $\lambda = 3\,\text{hrs}$ ([6, 2]).

external interactions	entities involved	MHC class involved
B phagocytosis of virus	B, HIV	–
MA phagocytosis of virus	MA, HIV	–
MA phagocytosis of IC	MA, IC	–
Interaction between TH and B	B, TH	class II
Interaction between TH and MA	TH, MA	class II
Interaction between Ab and HIV	Ab, HIV	–
Virus infection of MA	MA, HIV	–
Virus infection of DC	DC, HIV	–
Virus infection of TH	TH HIV	–
CTL lysis of MA	CTL, MA	class I
CTL lysis of DC	CTL, DC	class I
CTL lysis of TH	CTL, TH	class I

Table 1: Cell-to-cell interactions. Symbols are: B=lymphocyte B, TH=lymphocyte T helper or CD4 T, CTL=cytotoxic lymphocyte T or CD8 T, MA=macrophage, DC=dendritic cell, PLB=lymphocyte plasma B, IC=immune complexes or antibody-antigen binding.

Digestion and presentation	entity involved	MHC class involved
B digestion	B	class II
MA digestion	MA	class II
MA processing	MA	class I
TH processing	TH	class I
DC processing	DC	class I

Table 2: Cell-internal interactions: digestion and presentation on class I and II MHC of antigen peptides.

Differences in cells mobility are taken into account as well. TH cells are the fastest ones, with an average velocity of 11 $\mu m/min$, followed by B cells with 6 $\mu m/min$ and DC with a velocity of 3 $\mu m/min$ ([5]).

2 Setting the parameters

The parameters of the model can be classified in three categories: (1) unknown values (free parameters) which we set after a tuning procedure that starts with an initial guess based on empirical rules and iteratively improves by looking at the results of the tuning simulations; (2) parameters that correspond to the initial conditions of the system and that determine the problem under investigation; (3) parameters whose value is well known and available from immunology literature.

Setting the free parameters is a long process that required to run a high number of simulations for each parameter in order to get enough statistics. Parameter tuning is made by checking the results against well known clinical (or experimental) data. For instance, we tuned the parameters to achieve the following values:

- an average viral set point ($4.52 \div 0.80$);
- an acute phase of HIV lasting from three weeks to one year [1, 3];
- an elapsed time between infection and onset of AIDS of about $7 \div 12$ years in normal progressors [1];
- 5% of rapid progressors (*i.e.* infected people who progress to death for opportunistic diseases within 5 years after HIV-1 primary infection) [1];
- 12% of long term progressors (*i.e.* individuals who do not develop AIDS within 20 years) [1].

The first step in the tuning process is to select a wild-type HIV-1 that is represented by two bit strings (each l bits long), one corresponds to the epitope and the other to the peptide. In the simulations, the bit string length l is equal to 16 for a potential repertoire of 2^{16} distinct receptors and molecules. Actually, since the virus is represented by one epitope and a single peptide, each strain is identified by $2l = 32$ bits. So, the potential number of different virus strains becomes equal to 2^{32}.

The viral phenotype corresponding to the activation, replication, and mutation rate, is coded by a bit string. In particular we choose the string corresponding to 0.00066 mutation rate, 0.0011 activation rate, and 0.011 duplication rate.

The selection of the (only) viral peptide is driven by the experimental viral load set point equal to 4.5. We a select it as follows: first, we choose a binary string for the MHC that we pick from a set of binary strings, then the peptide having a certain affinity level with the chosen MHC molecules is selected.

In early infection, HIV is characteristically trophic for the CCR5 chemokine receptor on macrophages (monocytrophic). The emergence of mutated strains of the virus can lead to an increase of the virus binding to the CXCR4 chemokine receptor, conferring increased trophism for $CD4^+$ T cells (lymphotrophic) [4]. Therefore we consider the following values for the probability of the interaction of HIV-1 with macrophages, dendritic cells and TH cells: 0.2, 0.02 and 0.1, respectively. These selected values together with the chosen peptide result in a viral set point in line with clinical observations.

Another important parameter is the parameter expressing the efficiency of Interleukin-2 in stimulating the growth of the lymphocytes. It is inversely proportional to the ability of T cells to clone: by increasing it the T cells duplication reduces and the immuno-response is weakened. As a consequence the depletion of the T cells is stronger and patients die because the CD4 count falls below 50 cells/μl.

Finally, we tune the duration of the acute phase of the infection. This can be done by changing the half-life of MHC-peptide complexes expressed on the surface of Antigen Presenting Cells. An appropriate value for this parameter leads to an acute phase that lasts from 10 weeks (0.2 years) to one year.

References

[1] F Castiglione, F Poccia, G D'Offizi, and M Bernaschi. Mutation, fitness, viral diversity, and predictive markers of disease progression in a camputational model of hiv type 1 infection. *AIDS Res Hum Retrovirus*, 20(12):1314–1323, 2004.

[2] Karl Francis and Bernhard O Palsson. Effective intercellular communication distances are determined by the relative time constants for cyto/chemokine secretion and diffusion. *Proc. Natl. Acad. Sci. USA*, 94(23):12258–12262, 1997.

[3] Z Hel, J R McGhee, and J Mestecky. Hiv infection: first battle decides the war. *Trends Immunol*, 27(6):274–281, 2006.

[4] M Helbert and J Breuer. Monitoring patients with hiv disease. *J Clin Pathol*, 53(4):266–272, 2000.

[5] Mark J Miller, Sindy H Wei, Michael D Cahalan, and Ian Parker. T cell repertoire scanning is promoted by dynamic dendritic cell behavior and random t cell motility in the lymph node. *Proc. Natl. Acad. Sci. USA*, 101:998–1003, 2004.

[6] J L Segovia-Juarez, S Ganguli, and D Kirschner. Identifying control mechanisms of granuloma formation during m. tuberculosis infection using an agent-based model. *Journal of Theoretical Biology*, 231(3):357–376, 2004.
