# Supplementary material for: Timely HAART initiation may pave the way for a better viral control
Source: BMC Infect Dis. 2011 Mar 1;11:56. doi: 10.1186/1471-2334-11-56 (PMC3058048; doi:10.1186/1471-2334-11-56)
Supplement: Additional file 2 — Parameters tuning for the simulation of HIV infected virtual patients. This file reports details of the parameter setting and tuning. [file 1471-2334-11-56-S2.PDF]

Supplementary material S.2

Parameters tuning for the simulation of HIV infected virtual patients

Hereafter, we present the results of the simulations aimed at the tuning of the free parameters of the model. The tuning process is required to faithfully reproduce clinical and laboratory data (published elsewhere) that can be summarized in the statistics reported in Table 1. In particular the peak plasma viral load during the hyper acute phase is achieved within 8 weeks from the primary infection and correlate directly with post-set-point plateau levels of virus that is $\log_{10}(vRNA) \simeq 4.75$ copies/ml and is reached in 12 weeks. The width of the viremia peak, that is the time between the first antigen injection and the reaching of the plateau, corresponds to the so called acute phase. Another important parameter is the time between the infection and the onset of AIDS (median time in Table 1) in normal progressors. Clinical studies show that, in adults, this time is about 10 years. For what concerns the last two columns of Table 1, we discuss them in the end of this document.

In table 2 we report the results of the study carried out for the choice of the parameters. Each line is the output of thousand simulations and corresponds to a group of *virtual patient*. We start with a quasi-random choice (that is with a simple guess) of the free parameters (`Run 1`) and then we change the parameters that are colored in red in the table. Those that are shown in blu represent the parameters changed with respect to `Run 5`. The optimal setting (`Run 7`) is highlighted in yellow.

The first column of Table 2 represents the total number of simulations performed for each choice of the parameter set while the second one is the number of virus that is injected at the beginning of each simulation.

Another important parameter is `IL2eff` that is a factor expressing the efficacy of the Interleukin-2 in stimulating the growth of the lymphocytes. It is inversely proportional to the ability of T cells to clone: the increase of `IL2eff` reduces the T cells duplication and the immuno-response is weakened. As a consequence, the depletion of the T cells becomes more significant. We set this parameter in order to have a reasonable outcome that is about 80% of patient that die in 20 years from the first infection in absence of antiretroviral treatment; 20% that do not die in 20 years also in absence of therapy.

It is worth to stress that we map the phenotype of the virus by a triplet of numbers (p_w, p_r, p_m) between 0 and 1 that represent the activation, replication, and mutation rate of the virus. The range of possible values for (p_w, p_r, p_m) is chosen by looking at the results of a set of preliminary runs in order to reproduce, on average, the three-phase dynamics of HIV infection. A suitable set of initial values for the triplet (p_w, p_r, p_m) is required. We set them in order to give a reasonable acute phase no longer then a few weeks, with the virus load decreasing considerably. The wild-type HIV-1 is represented as just one peptide and one epitope. Changing either the peptide or/and the epitope produces different outcomes: 1) if the peptide is modified, the affinity with the molecules MHC class I or II changes; 2) if the epitope is modified, then one of the three values of the triplet (p_w, p_r, p_m) is modified. Thus we fix a random value for the peptide and then we choose suitable strings for MHC class I and II able to have high affinity with the virus.

Fixing MHC-I and MHC-II leads to a viral set point in good agreement with the experiments but gives a highly deterministic histogram for the "time to AIDS". Thus we allow the MCH strings to flip some bits, as shown in the forth column of Table 2 that specifies how many bits are changed from the selected MHCs. If MHCs are randomly chosen (`Run 6`: all bits can flip) a log-normal distribution works well in fitting the "time to AIDS" histogram but some other experimental values are not recovered (*e.g.,* in table 4 the number of "`Ag is defeated`" indicating simulations not biologically relevant, is too high).

The parameter `pRemove` represents the probability that a virus that has been phagocyted by an antigen presenting cell degrades before the digestion and presentation to the MHC molecule takes place. A high value of translates in a high viral particles consumption, or, in other words, in a high virus clearance rate from APCs. Since this parameters influences the duration of the immune response, we set its value to obtain an immune response within 1 year of infection. To summarize, during the first years we have a peak in the $CD4^+$ T cells count with consequent cell decrease due to the destructive action of the virus.

The parameter μl is the system size in micro-liters of peripheral blood. By increasing it, we obtain a more realistic description of the HIV infection. The simulator is constructed in a way to allow a good scaling of the results with respect to simulated volume.

Finally, P_w is the activation probability of the virus that we discussed above. Low values of this parameter may be interpreted either as strains having a poor adaptation or as strains that are activated very late in time. Also this parameter can affect the duration of the immune response.

In absence of antiretroviral treatment the time between primary infection in adults and the development of AIDS is well fitted by a log-normal distribution [1] centered around the clinical data of about 10 years. In table 3 we report the parameters of probability distribution function that fit our outcomes. In table 4 we report the results of each simulation. In particular in the first three columns we show the percentage of the possible outcomes of our runs: 1) Ag limit reached means the run terminated because of a too high antigen concentration; 2) TH< 50 means the patient dies for an opportunistic disease because the CD4$^+$ T cells count is too low; 3) Normal termination means that the patient is still alive at the end of simulation; 4) Ag is defeated is the less realistic termination because it means that the virus is defeated.

Table 1: Experimental data.

Viral set point ($\log_{10}$)	HIV hyper acute phase (weeks)	HIV acute phase (weeks)	Median[1] (years)	Rapid[2] prog. (%)	Long[3] prog. (%)
4.5[1]	8 [2]	12[2]	7 $\div$ 12 [1]	5 [1]	12 [1]

In Fig. 1 we report the result of five-thousand simulations for the best set of runs that is colored in yellow in table 2. In panel (a) the histogram represents the distribution of the time between the primary infection and the onset of AIDS. The histogram can be fitted by a log-normal distribution with median of about 10 years in good agreement with clinical data (Muñoz *et al.* cited after [1]); in panel (b) we show how the runs are distributed with respect to the four possible termination conditions; in panel (c) the average of viral load as function of the time to AIDS. It shows a negative correlation as observed in reality; in panel (d) we show the results of the simulation of the first six months from the primary infection that correspond to the clinical acute phase. The peak of the plasma viral load during the hyper acute phase (grey box in the figure) is achieved within 8 weeks from the infection and correlate directly with post-set-point plateau levels of virus that is $\log_{10}(vRNA) \simeq 4.75 copies/ml$ and is reached in 12 weeks. All these data are in good agreement with clinical observations [2]. The immune system responds in the first 10 days from infection and the long-lasting depletion of the CD4$^+$ T cells starts after 10 weeks from infection. In absence of antiretroviral treatments, the time between infection in adults and the development of AIDS is approximately 7-12 years in normal progressor (median time is about 10 years) [1]. Long progressors (fifth column of the table4) proceed to the immune deficiency status in a longer time period. Clinical studies report that 12% of individuals do not develop AIDS within 20 years [1]. We simulate about 20 years and estimate that 12.80% (`Run 7`) of the virtual patients do not develop AIDS within this time interval.

The definition of Rapid Progressor (RP) is still a matter of debate. Anzala and coworkers define RP, a small percentage of HIV-infected individuals that rapidly progress to AIDS within four years after primary HIV-infection [3]. Indeed some individuals have been known to progress to AIDS and death within a year after prime-infection. Moreover, rapid progression was originally thought to be continent specific, as some studies reported that disease progression is more rapid in Africa [4, 3, 5] but other studies disagree about this view [6, 7].

[1] The time between infection in adults and the development of AIDS in normal progressor.

[2] Rapid progressor: infected people which progress to death for opportunistic disease within 5 years after HIV-1 primary infection.

[3] Long progressor: individuals which do not develop AIDS within 20 years.

[4] This is the average value of the viral load in the first 7.3 years.

Table 2: Input data

	Total runs	NmaxVirus	IL2eff	genMHC	Timesteps	pRemoveAg	μl	p_w
Run 1	1064	500	150	2	30000	0.3	3	0.3
Run 2	1069	100	250	3	30000	0.3	3	0.3
Run 3	1625	100	500	3	30000	0.3	3	0.3
Run 4	955	100	1000	3	30000	0.3	3	0.3
Run 5	1097	100	1000	3	21900	0.3	3	0.3
Run 6	693	100	1000	random	21900	0.3	3	0.3
Run 7	5000	100	1000	3	21900	0.6	3	0.3
Run 8	888	100	1000	3	21900	0.6	10	0.3
Run 9	1498	100	1000	3	21900	0.3	3	0.1
Run 10	1099	100	1000	3	21900	0.3	3	0.06

Table 3: Log-normal fit

	Bin	m	s	Mean	σ	Median
Run 1	34	2.46046	0.33520	12.38690	18.03098	11.71020
Run 2	40	2.41946	0.37983	12.08056	17.73499	11.23980
Run 3	24	2.26986	0.24691	9.97759	14.33048	9.67803
Run 4	28	2.33765	0.30564	10.85209	15.71838	10.35685
Run 5	17	2.29178	0.18784	10.06865	14.36650	9.89257
Run 6	10	2.46498	0.19959	11.99988	17.14195	11.76322
Run 7	15	2.32755	0.17894	10.41823	14.85297	10.25277
Run 8	12	2.34277	0.15792	10.54071	15.00063	10.41008
Run 9	17	2.23859	0.22308	9.61637	13.77198	9.38005
Run 10	24	2.26449	0.28179	10.01605	14.45452	9.62619

Table 4: Output data

	Ag limit reached (%)	TH<50 (%)	Normal term. (%)	Ag is defeated (%)	Viral set point [4]	Long prog. (%)
Run 1	62.68	18.70	16.16	0.00	4.60 ÷ 5.20	17.19
Run 2	60.14	29.84	9.35	0.37	4.60 ÷ 4.90	13.75
Run 3	45.66	41.84	11.93	0.95	4.60 ÷ 4.80	15.50
Run 4	34.55	50.47	14.34	0.41	$\simeq$ 4.60	14.55
Run 5	18.96	59.34	21.60	0.09	$\simeq$ 4.75	11.85
Run 6	11.39	34.63	36.36	17.60	$\simeq$ 4.25	18.61
Run 7	13.76	64.96	21.18	0.10	$\simeq$ 4.75	12.80
Run 8	30.74	38.06	31.19	0.00	$\simeq$ 4.90	16.66
Run 9	2.40	80.57	17.02	0.00	$\simeq$ 4.50	8.41
Run 10	1.09	83.43	15.46	0.00	$\simeq$ 4.30	9.09

Here we consider as RP the infected people that progress to death for opportunistic diseases within 5 years after HIV-1 primary infection. We simulate this situation by injecting during the third year from primary

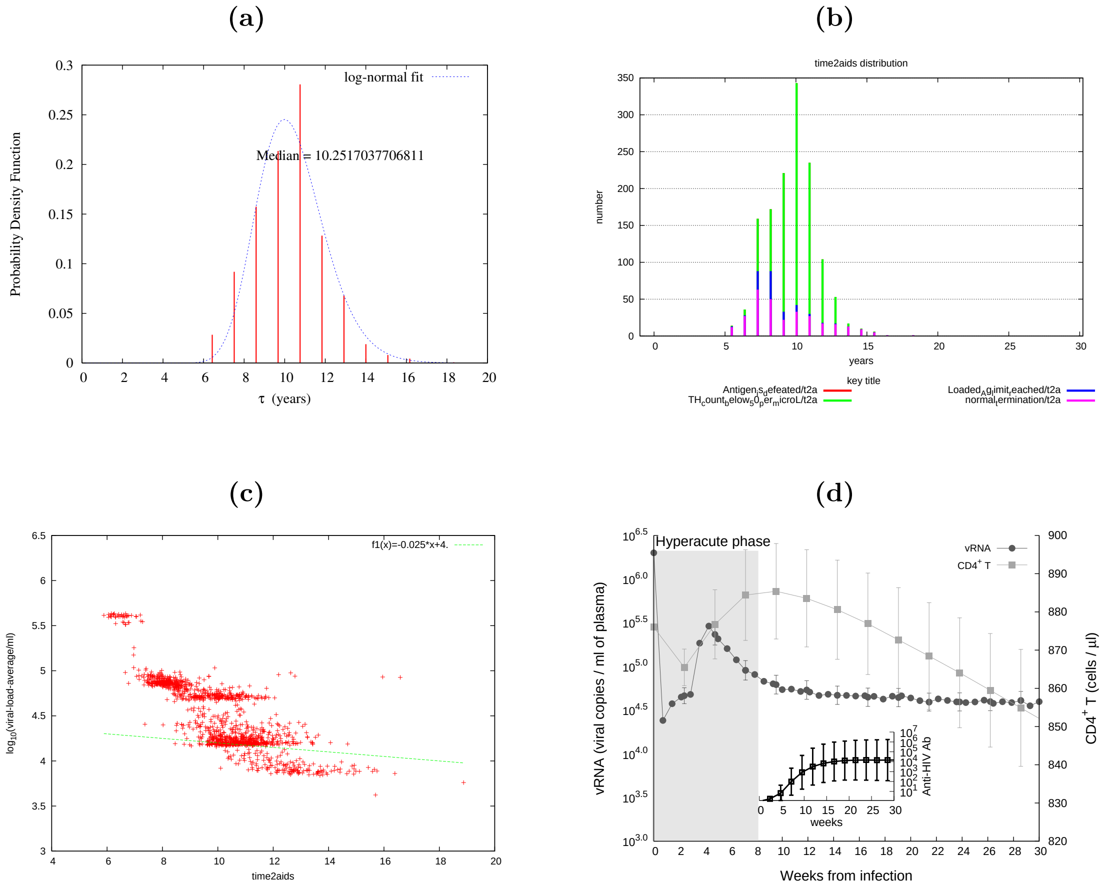

Figure 1: RUN 7. Panel (a) shows the probability distribution of the time between infection in adults and the development of AIDS in normal progressor. The dashed line corresponds to a fit with a log-normal function. In panel (b), on top of the same histogram, we show the relative proportion of runs that have terminated for one of the four possible termination conditions: (1) the antigen is defeated; (2) the level od TH cells goes very low; (3) the spread of the virus is too large; (4) the run terminates normally. In panel (c) the average viral load as function of the time to AIDS as observed in reality. Panel (d) shows the average of the viral set point and of the CD4$^+$ T cells count within the first 30 weeks from infections. See text for detailed discussion.

HIV-1 infection an uninfective bacterium that is complementary, in terms of bits, to the HIV-1 wild-type. In 1000 runs we have that 49 end within 5 years from primary HIV-1 infection for opportunistic disease. It means that we have 4.9% of rapid progressors in good agreement with the laboratory results shown in Table 1. It is worth to highlight that we consider RP as a random variable following a binomial distribution with probability $p = 49/1000$ (more precisely this is the frequency of the event that tends towards to the true probability when $N \to \infty$ and the error in this assumption is equal to $\sigma/\sqrt{N}$ where σ is the standard deviation of the distribution), mean value $Np = 49$ (N is the number of simulations) and standard deviation $\sigma = \sqrt{(Np(1-p))} \simeq 7$. In the upper panel of figure 2 we plot the infection with a bacterium to show the occurrence of RP and in the lower panel the survival curves corresponding to the tuning runs of Table 4 where the bacterium injection was absent. In this latest situation RP can be thought only as infected people that develop AIDS within early years of primary HIV-1 infection. We can see that in the optimal `Run 7`

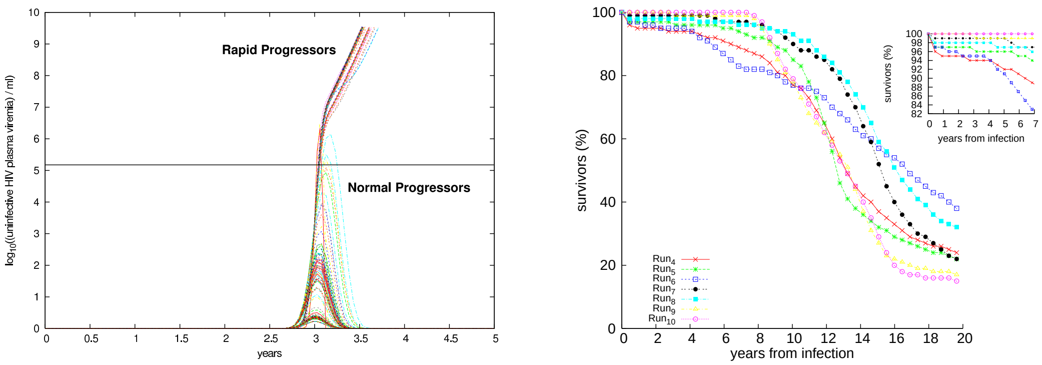

Figure 2: Uninfective HIV plasma viremia (left panel) in 1000 runs with the further injection of a bacterium. In the right panel we plot the survival curves referring to the above runs in absence of bacterium and antiretroviral therapy.

(black filled circles) we obtain the clinical value of 5% only within 9-10 years from infection.

References

[1] Castiglione F, Poccia F, D'Offizi G, Bernaschi M: **Mutation, fitness, viral diversity, and predictive markers of disease progression in a computational model of HIV type 1 infection**. *AIDS Res Hum Retroviruses* 2004, **20**(12):1314–23.

[2] Hel Z, McGhee JR, Mestecky J: **HIV infection: first battle decides the war**. *Trends in Immunology* 2006, **27**(6):274–281.

[3] Anzala A, Nagelkerke J, Bwayo J, Holton D, Moses S, Ngugi E, Ndinya-Achola J, Plummer F: **Rapid progression to disease in African sex workers with human immunodeficiency virus type 1 infection**. *J. Infect. Dis.* 1995, **171**:686–689.

[4] B N, Ryder R, Bila K, Mwandagalirwa K, Colebunders R, Francis H, TC JM, Quinn: **Human immunodeficiency virus infection among employees in an African hospital**. *N. Eng. J. Med.* 1988, **319(17)**:1123–1127.

[5] Whittle H, Egboga A, Todd J, Corrah T, Wilkins A, Demba E, Morgan G, Rolfe M, Berry N, Tedder R: **Clinical and laboratory predictors of survival in Gambian patients with symptomatic HIV-1 or HIV-1 infection**. *AIDS* 1992, **6(7)**:685–689.

[6] Marlink R, Kanki P, Thior I, Travers K, Eisen G, Siby T, Traore I, Hsieh C, Dia M, Gueye E: **Reduced rate of disease development after HIV-2 infection as compared to HIV-1**. *Science* 1994, **265(5178)**:1587–1590.

[7] French N, Mujugira A, Nakiyingi J, Mulder D, Janoff E, Gilks C: **Immunologic and clinical stages in HIV-1-infected Ugandan adults are comparable and provide no evidence of rapid progression but poor survival with advanced disease**. *J. Acquir Immune Defic. Syndr.* 1999, **22**:509–516.
